# The plasma separation card as a novel solution for enhancing central laboratory capability for HIV-1 viral load monitoring in limited-access settings

**DOI:** 10.1371/journal.pgph.0002099

**Published:** 2023-06-28

**Authors:** Charles Kiyaga, Caroline Makoha, Ivan Nkugwa, Christopher Okiira, Richard Okwir, Sirak Zenebe Gebreab, Patricia Rodriguez-Ventosa Suarez, Benjamin LaBrot, Ana Carrasco Durán

**Affiliations:** 1 Uganda National Health Laboratory Services, Kampala, Uganda; 2 Kisenyi Health Center IV, Kampala, Uganda; 3 Roche Diagnostics International AG, Rotkreuz, Switzerland; 4 Roche Molecular Systems, Pleasanton, CA, United States of America; PLOS: Public Library of Science, UNITED STATES

## Abstract

Measurement of HIV-1 viral load (VL) is essential for monitoring antiretroviral treatment (ART) efficacy. The preferred specimen type for VL is plasma, but in remote settings where collection and preservation of plasma many not be possible, dried blood spots (DBS) are often used instead. A new specimen collection matrix, the cobas plasma separation card (PSC, Roche Diagnostics Solutions), enables specimen preparation from a finger prick or venous blood, using a multi-layer absorption and filtration design that results in a specimen similar to dried plasma. We sought to confirm the correlation between VL results obtained using PSC prepared from venous blood to those from plasma or DBS, as well as PSC prepared with capillary blood from a finger prick. PSC, DBS and plasma were prepared with blood from HIV-1 infected persons attending a primary care clinic in Kampala, Uganda. VL in PSC and plasma was measured using cobas HIV-1 (Roche Diagnostics), while VL in DBS was measured with RealTime HIV-1 (Abbott Diagnostics). The correlation between VL from plasma and PSC made from capillary or venous blood was high (regression coefficient of determination r^2^ between 0.87 and 0.91), and there was good agreement based on mean bias (-0.14 to 0.24 log_10_ copies/mL) and classification of VL above or below 1000 copies/mL (91.4% agreement). In contrast, VL from DBS was lower than plasma or PSC (mean bias 0.51 to 0.63 log_10_ copies/mL) and not as well correlated (r^2^ 0.78 to 0.81, 75.1–80.5% agreement). These results confirm the utility of PSC as an alternative specimen type for HIV-1 viral load measurement in areas where preparation and optimal storage or shipment of plasma is an obstacle to provision of treatment and care of HIV-1 infected people.

## Background

Human immunodeficiency virus (HIV) is the etiologic agent of acquired immunodeficiency syndrome (AIDS). There are an estimated 38 million people infected with HIV-1 worldwide, nearly 29 million of whom are receiving anti-retroviral therapy (ART) [1]. Viral load (VL) testing to measure the amount of HIV-1 RNA in blood is an essential tool for monitoring of disease progression and response to ART.

The preferred clinical specimen for HIV-1 viral load testing is plasma. Collection, storage and shipment of plasma specimens can be challenging, especially in remote or resource-limited settings, since phlebotomy and centrifugation are needed to prepare the plasma, and refrigeration is required to prevent the degradation of HIV-1 RNA. A high proportion of people living with HIV infection reside in Africa, where basic health services such as phlebotomy and a reliable cold chain remain difficult to access. An alternative specimen type is dried blood spots (DBS), which can be prepared from capillary blood via finger or heel prick or from whole blood collected by venipuncture [2]. Once dried, HIV-1 RNA is relatively stable in DBS at ambient temperature for a limited time but is sensitive to humidity and so must be stored desiccated. In some settings, amplification rates are sub-optimal [3]. Since infected lymphocytes are present in the DBS, there is potential for contribution from integrated proviral DNA, or cellular viral RNA, to the viral load result, including over-estimation and false positives especially at low VL [4–6]. This interference can be mitigated by the use of nucleic acid extraction methods that are selective for RNA [7, 8], quantitation methods that are specific for RNA [9, 10], or elution of virus particles from the DBS [11, 12]. The use of DBS in conjunction with the Abbott RealTimeHIV VL test is a viable and available option in low- and middle-income countries [7, 8].

The cobas Plasma Separation Card (PSC) is a recently developed specimen collection and storage matrix that has the potential to expand access to HIV VL testing [13]. Dried and stabilized plasma spots can be prepared without need for trained phlebotomists or a centrifuge, and stored or transported at ambient temperature while maintaining RNA integrity for longer periods of time than traditional methods [13, 14]. The use of dried plasma from PSC avoids cell-associated HIV-1 nucleic acids that may lead to over quantification of VL.

Studies that compare VL from DBS prepared from finger prick capillary blood to DBS or plasma from blood collected by venipuncture (venous blood) [8, 9, 15–21] have demonstrated acceptable performance in terms of correlation to VL measured in plasma using the same method, and good agreement in classification of VL above or below 1000 copies/mL (the WHO-recommended threshold used to define treatment failure when using DBS as the specimen type for viral load testing) [22], albeit with some loss of signal and decreased sensitivity. Similar comparisons of finger prick to venous blood using PSC have not been reported previously.

The present study was designed to evaluate the correlation of HIV-1 VL obtained using PSC made from capillary and venous blood using the Roche cobas HIV-1 test. We also sought to confirm the correlation between VL results from PSC and plasma, prepared under field conditions. We compared VL results obtained with PSC to those from DBS tested with the Abbott RealTime HIV-1 test. Finally, the repeatability of VL measurements from PSC (made from capillary and venous blood) with cobas HIV-1 and DBS with RealTime HIV-1 was assessed through triplicate testing of selected specimens.

## Materials and methods

### Ethics statement

Prospectively collected samples were delinked from patient identifying information by labeling with a study identifier. Before study initiation, the Principal Investigator and Roche obtained the approval of the Uganda National Health Laboratory Services (UNHLS) Research and Ethics Committee and the Uganda National Council for Science & Technology (UNCST).

### Study subjects and ethics

Participants were recruited from amongst attendees at the Kisenyi Health Center IV (KHCIV) HIV care clinic in Kampala, Uganda in two phases. The overall objective was to have 50 participants with VL in each category (Table 1). Consenting and sample collection was performed by health care professionals according to standardized procedures established at the site and following training by Roche. From June 2021 to September 2021, all consenting clinic attendees meeting the basic eligibility requirements (HIV-1 positive, age over 18 years, and VL test indicated) were included. When the target number of participants in the first group (VL undetectable) was reached (September 2021), clinic staff targeted patients who were more likely to have detectable VL, i.e. those who were newly diagnosed, previously untreated, had a previous detectable VL, or were suspected to be non-adherent to treatment. The targets of 50 participants per group were only reached for groups 1 and 5, mostly due to limitations related to the COVID-19 pandemic, and enrolment was halted in April 2022. Patients with undetectable VL (group 1) and high VL (group 5) who were enrolled, even though the target of 50 participants per group had been reached, became part of the repeatability study (see below). Only results from the standard EDTA plasma specimens, collected for routine determination of HIV-1 VL, were used for patient management.

**Table 1:** Groups of specimens according to viral load in plasma.

Group	HIV-1 plasma viral load (copies/mL)	N
1	Target Not Detected or < LLOQ	50
2	≥ 790 (LLOQ) to < 1000	8
3	≥ 1000 to < 5000	31
4	≥ 5000 to < 20,000	46
5	≥ 20,000	50
	**Total**	**185**

### Specimen collection and storage

Capillary blood was collected from a finger prick as described in the PSC Method Sheet (Roche Diagnostics Solutions) and used to create three spots of 140 μL each on the capillary PSC for each patient using capillary tubes at KHCIV. The components of the PSC collection kit are shown in Fig A in S1 Data. Each capillary PSC was dried for a minimum of four hours, transferred to a sample bag with 4 g desiccant, then placed in a foiled transport bag for shipping to UNHLS at ambient temperature (up to 45°C and 85% relative humidity) within 48 hours. At UNHLS, the specimens (“capillary PSC”) were stored at 2–8°C until testing. Blood samples (10 mL) were collected in EDTA anti-coagulant at KHCIV and transported to UNHLS on the same day for preparation of PSC and DBS from venous blood, followed by plasma separation. Blood was stored at 2–8°C for less than 24 hours before sample preparation; spotting and plasma separation. Three PSC spots of 140 μL were prepared from the EDTA whole blood, dried for a minimum of four hours and stored in a sample bag with 4 g desiccant at 2–8°C before testing (“venous PSC”). DBS were prepared by pipetting 70 μL of blood onto five spots on BioSample TFN CE perforated filter paper cards. DBS cards were dried for a minimum of four hours and stored in a sealed plastic bag with two to three desiccant packs and stored at 2–8°C. After spotting PSC and DBS, residual whole blood was centrifuged for plasma separation.

Repeatability of VL test results was evaluated using PSC (capillary and venous) as well as DBS from the same patient, tested in triplicate (i.e. using three different spots on the cards) at the same time and on the same instrument.

### Viral load testing

HIV-1 VL assays were performed at UNHLS by trained local staff. The tests used for the different specimen types were as follow:

- PSC from finger prick (capillary blood), tested with cobas HIV-1 (“capillary PSC”)
- PSC from EDTA blood (venous blood), tested with cobas HIV-1 (“venous PSC”)
- DBS from EDTA blood (venous blood), tested with RealTime HIV (“DBS”)
- plasma from EDTA blood (venous blood), tested with cobas HIV-1

cobas HIV-1 tests were performed according to the manufacturer’s instructions for the cobas 8800 instrument (Roche Diagnostics) using PSC (limit of detection or LOD: 790 copies/mL) or plasma (200 μL volume protocol, LOD: 50 copies/mL) [23]. RealTime HIV-1 tests were performed on DBS using one DBS spot on the m2000 instrument according to the manufacturer’s instructions (Abbott Diagnostics, Des Plaines, IA, USA; LOD: 839 copies/mL) [8, 24].

### Data analysis

Undetectable VL results were assigned an arbitrary value of 0.5 log_10_ copies/mL, and results between the LOD and LLOQ were assigned an arbitrary value of half the LLOQ, for illustrative purposes only. Confidence intervals were calculated using the Wilson score method.

## Results

Four types of specimen were used for VL testing: capillary and venous PSC, DBS, and plasma. Five pairwise comparisons were performed: capillary PSC, venous PSC, and DBS were compared to plasma, and venous PSC was compared to capillary PSC and DBS.

### 1. Comparison of VL in capillary PSC and plasma

**Fig 1:**
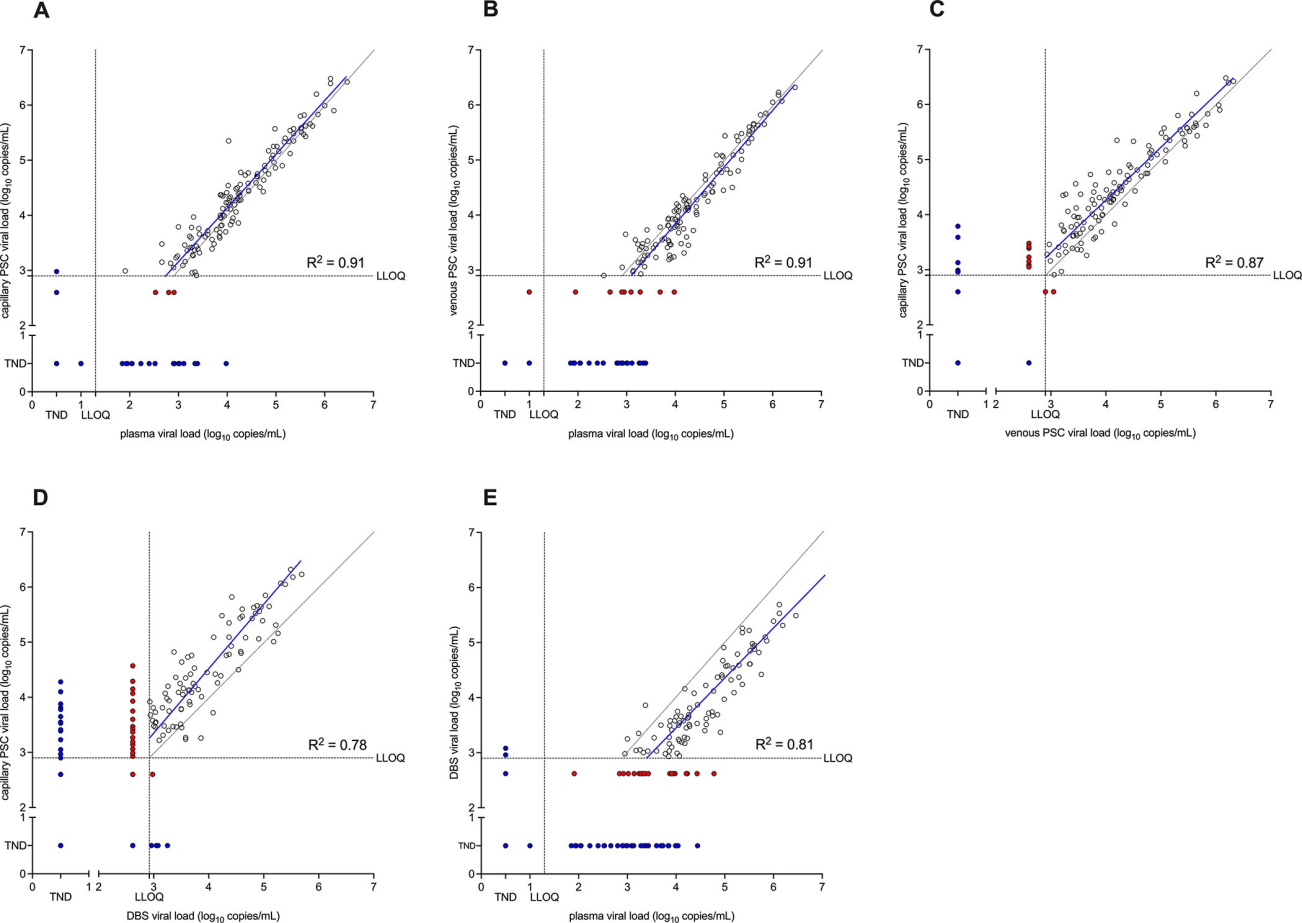
Deming regression plots of VL measurements from different specimen types. A: capillary PSC vs. plasma. B: venous PSC vs. plasma. C: capillary PSC vs. venous PSC. D. capillary PSC vs. DBS. E. DBS vs. plasma. Filled red circles indicate one or both results < LLOQ but > LOD. Filled blue circles indicate one or both results < LOD. Regression lines (blue) and correlation coefficients (R^2^) were calculated based only on results >LLOQ for both assays.

**Fig 2:**
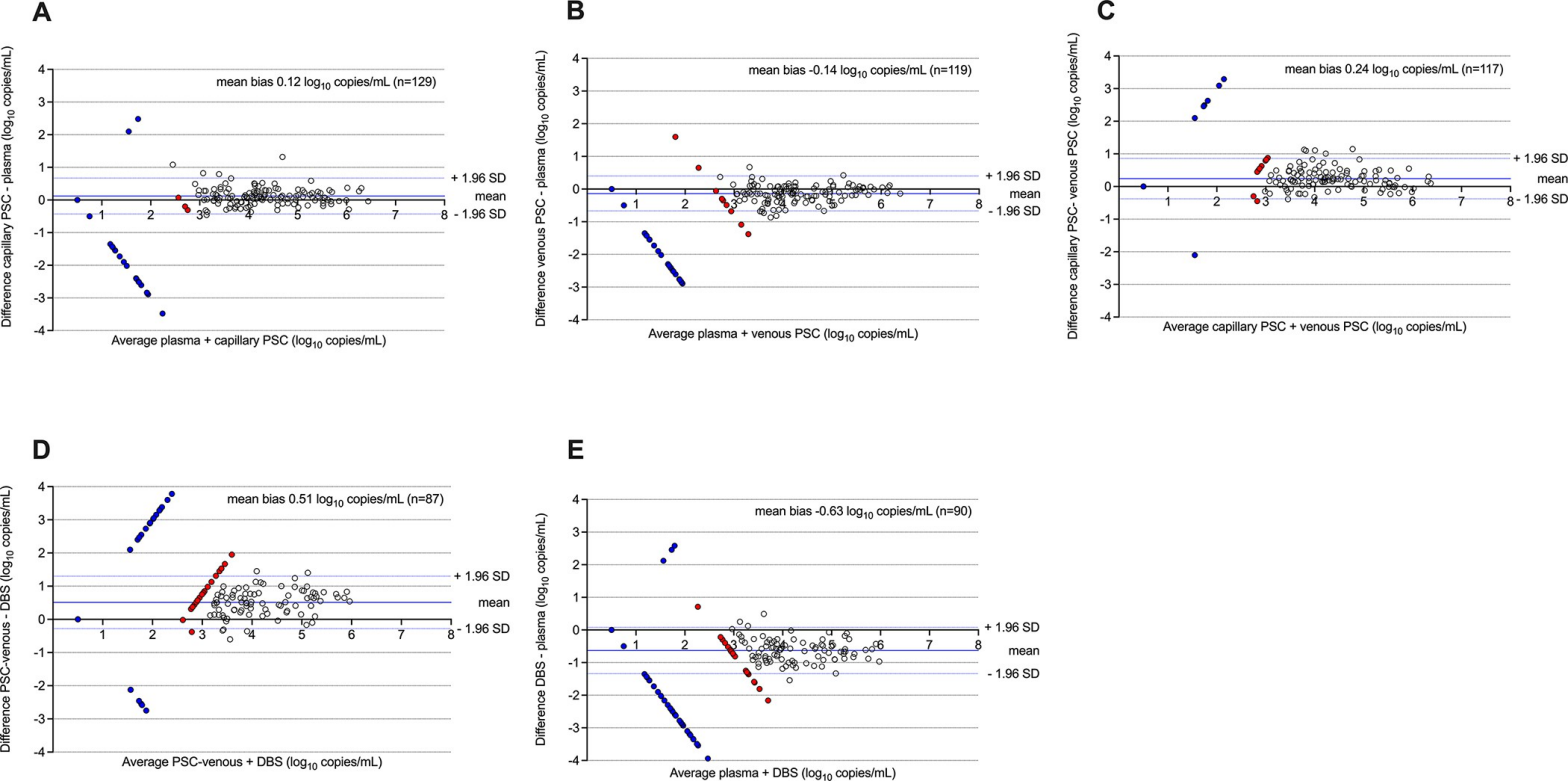
Bland-Altman plots of VL measurements from different specimen types. A: capillary PSC vs. plasma. B: venous PSC vs. plasma. C: capillary PSC vs. venous PSC. D. capillary PSC vs. DBS. E. DBS vs. plasma. Filled red circles indicate one or both results < LLOQ but > LOD. Filled blue circles indicate one or both results < LOD. Mean bias (solid blue line) and 1.96 SD above and below the mean (encompassing 95% of the data in the linear range of both assays; dotted blue lines) were calculated based only on results >LLOQ for both assays. The diagonal solid black lines represent exact correspondence of the two measurements. Dotted black lines indicate the LLOQ of the assay.

The correlation between VL results that were above the LLOQ for both measurements from PSC prepared using capillary blood from finger prick or plasma from venous blood was high (r^2^ = 0.91; Fig 1A). The mean bias (Capillary PSC–plasma) was 0.12 log_10_ copies/mL (95% CI: 0.07 to 0.17 log_10_ copies/mL; Fig 2A). There were two results with a difference greater than 1 log_10_ copies/mL, both of which were higher in Capillary PSC (range: 1.07–1.32 log_10_ copies/mL; Table A in S1 Data).

Classification of VL above or below the 1000 copies/mL threshold demonstrated 91.4% overall agreement (95% CI: 87.0 to 95.0%; Table 2). Of the 16 discordant pairs of results, nine were above the threshold in plasma (thus representing downward misclassification by Capillary PSC; the other seven are upward misclassification). The most extreme case of discordance had Capillary PSC VL of 6122 copies/mL and 999 copies/mL in plasma. Another case had undetectable Capillary PSC VL and 9645 copies/mL in plasma (Table B in S1 Data).

**Table 2:** Concordance analysis: HIV-1 VL in capillary PSC vs. plasma.

		plasma		
		<1000 copies/mL	≥1000 copies/mL	Total
**capillary PSC**	**<1000 copies/mL**	51	9	60
	**≥1000 copies/mL**	7	118	125
	**Total**	58	127	185
**Overall Percent Agreement (95% CI)**		91.4% (86.4%, 94.6%)		

CI = Confidence Interval; PSC = Plasma Separation Card

### 2. Comparison of VL in venous PSC and plasma

The correlation between VL results that were above the LLOQ for both measurements from PSC prepared using venous blood following venipuncture or plasma from venous blood was high (r^2^ = 0.91; Fig 1B). The mean bias (Venous PSC–plasma) was -0.14 log_10_ copies/mL (95% CI: -0.18 to -0.09 log_10_ copies/mL; Fig 2B). There were no results with a difference greater than 1 log_10_ copies/mL.

Classification of VL above or below the 1000 copies/mL threshold demonstrated 91.4% overall agreement (95% CI: 87.0–95.0%; Table 3). Of the 16 discordant pairs of results, 14 were above the threshold in plasma (thus representing predominantly downward misclassification by Venous PSC). The most extreme case of discordance had detectable Venous PSC VL below the LLOQ and 9645 copies/mL in plasma. Another case had Venous PSC VL of 4445 copies/mL and 952 copies/mL in plasma (Table B in S1 Data).

**Table 3:** Concordance analysis: HIV-1 VL in venous PSC vs. plasma.

		plasma		
		<1000 copies/mL	≥1000 copies/mL	Total
**venous PSC**	**<1000 copies/mL**	56	14	70
	**≥1000 copies/mL**	2	113	115
	**Total**	58	127	185
**Overall Percent Agreement (95% CI)**		91.4% (86.4%, 94.6%)		

CI = Confidence Interval; PSC = Plasma Separation Card

### 3. Comparison of VL in capillary PSC and venous PSC

The correlation between VL results that were above the LLOQ for both measurements from PSC prepared using capillary blood from finger prick or venous blood following venipuncture was high (r^2^ = 0.87; Fig 1C). The mean bias (Capillary PSC–Venous PSC) was 0.24 log_10_ copies/mL (95% CI: 0.18 to 0.30 log_10_ copies/mL; Fig 2C). There were five results with a difference greater than 1 log_10_ copies/mL, all of which were higher in Capillary PSC (range: 1.00–1.14 log_10_ copies/mL; Table A in S1 Data).

Classification of VL above or below the 1000 copies/mL threshold demonstrated 91.4% overall agreement (95% CI: 87.0 to 95.0%; Table 4). Of the 16 discordant pairs of results, 13 were above the threshold in Capillary PSC. The most extreme case of discordance had Capillary PSC VL of 6122 copies/mL and undetectable VL in Venous PSC (Table B in S1 Data).

**Table 4:** Concordance analysis: HIV-1 VL in capillary PSC vs. venous PSC.

		PSC- capillary		
		<1000 copies/mL	≥1000 copies/mL	Total
**PSC- venipuncture**	**<1000 copies/mL**	57	13	70
	**≥1000 copies/mL**	3	112	115
	**Total**	60	125	185
**Overall Percent Agreement (95% CI)**		91.4% (86.4%, 94.6%)		

CI = Confidence Interval; PSC = Plasma Separation Card

### 4. Comparison of VL in venous PSC and DBS

The correlation between VL results that were above the LLOQ for both measurements from PSC or DBS from venous blood was moderate (r^2^ = 0.78; Fig 1D). The mean bias (Venous PSC–DBS) was 0.51 log_10_ copies/mL (95% CI: 0.43 to 0.60 log_10_ copies/mL; Fig 2D). There were six results with a difference greater than 1 log_10_ copies/mL, all of which were higher in Venous PSC (range: 1.07–1.45 log_10_ copies/mL; Table A in S1 Data).

Classification of VL above or below the 1000 copies/mL threshold demonstrated 80.5% overall agreement (95% CI: 74.2 to 85.6%; Table 5). Of the 36 discordant pairs of results, 33 were above the threshold in Venous PSC. The most extreme case of discordance had Venous PSC VL of 36,828 copies/mL and detectable but below LLOQ in DBS (Table B in S1 Data). Of 93 samples with VL < LLOQ in DBS, 32 had VL in Venous PSC over the LLOQ, and nine had VL over 10,000 copies/mL.

**Table 5:** Concordance analysis: HIV-1 VL in venous PSC vs. DBS.

		DBS		
		<1000 copies/mL	≥1000 copies/mL	Total
**PSC- venous**	**<1000 copies/mL**	67	3	70
	**≥1000 copies/mL**	33	82	115
	**Total**	100	85	185
**Overall Percent Agreement (95% CI)**		80.5% (74.2%, 85.6%)		

CI = Confidence Interval; PSC = Plasma Separation Card

### 5. Comparison of VL in DBS and plasma

The correlation between VL results that were above the LLOQ for both measurements from DBS prepared using venous blood following venipuncture or plasma from venous blood was moderate (r^2^ = 0.81; Fig 1E). The mean bias (DBS–plasma) was -0.63 log_10_ copies/mL (95% CI: -0.71 to -0.56 log_10_ copies/mL; Fig 2E). There were 13 results with a difference greater than 1 log_10_ copies/mL, all of which were higher in plasma (range: 1.00–1.54 log_10_ copies/mL; Table A in S1 Data).

Classification of VL above or below the 1000 copies/mL threshold demonstrated 75.1% overall agreement (95% CI: 68.4–80.8%; Table 6). Of the 46 discordant pairs of results, 44 were above the threshold in plasma (thus representing predominantly downward misclassification by DBS). The most extreme case of discordance had detectable DBS VL below the LLOQ and 60,115 copies/mL in plasma (Table B in S1 Data).

**Table 6:** Concordance analysis: HIV-1 VL in DBS vs. plasma.

		DBS		
		<1000 copies/mL	≥1000 copies/mL	Total
**plasma**	**<1000 copies/mL**	56	2	58
	**≥1000 copies/mL**	44	83	127
	**Total**	100	85	185
**Overall Percent Agreement (95% CI)**		75.1% (68.4%, 80.8%)		

CI = Confidence Interval; PSC = Plasma Separation Card

The observation that VL from DBS were approximately 0.5 log_10_ copies/mL lower than from plasma or PSC prompted us to investigate likely causes. Due to logistical constraints at the testing site, the PSC, DBS and plasma from each patient were not tested at the same time, leading to the potential for a difference in storage time between specimen preparation and VL testing. The average time was 26 days (interquartile range, IQR: 16–37 days; range: 4 to 56 days) for capillary PSC, 25 days (IQR: 15–36 days; range: 3 to 52 days) for venous PSC, and 42 days (IQR: 24–50 days; range: 7 to 109 days) for DBS. However, there was no association between storage time, or difference in storage time between DBS and venous PSC from the same patient, and the difference in VL (see Fig B in S1 Data).

### Repeatability

**Fig 3:**
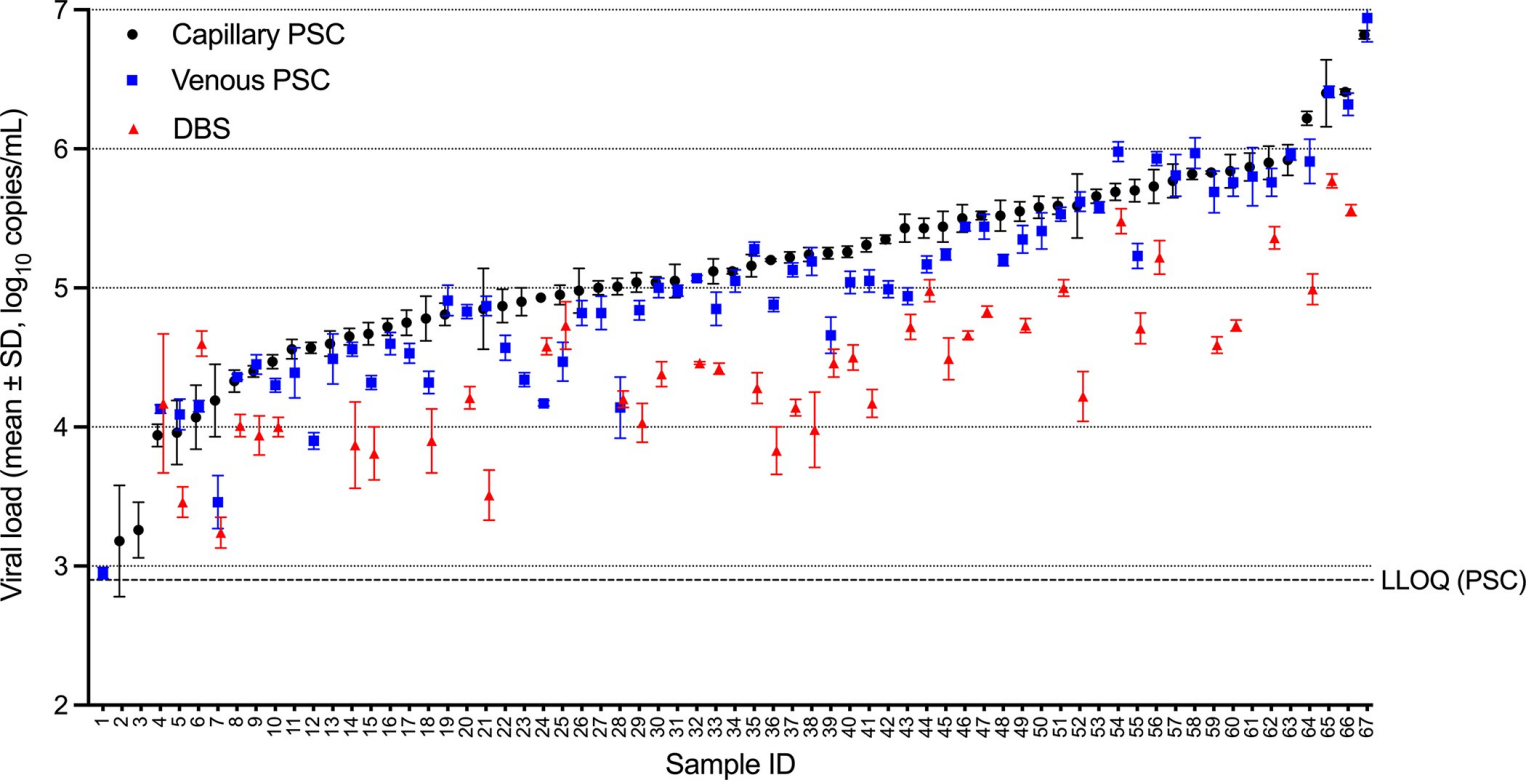
Repeatability of VL measurements from different specimen types. The mean and +/- SD for repeated VL measurements from capillary PSC (black circles), venous PSC (blue squares), and DBS (red triangles) are plotted on the Y axis (arbitrary sample ID) in order of increasing capillary PSC VL (or venous PSC if fewer than two valid results were available for capillary PSC).

There were 62, 64 and 43 samples with at least two valid replicate results for capillary PSC, venous PSC, and DBS, respectively. An instrument run failure led to missing data for several DBS specimens. The mean and median SD and %CV were similar for all three specimen types (Table 7). The mean VL with SD for the valid results from the three specimen types are shown in Fig 3. The mean VL followed the same pattern as described above for the single replicate comparisons: VL from DBS was on average 0.57 log_10_ copies/mL lower in DBS vs venous PSC, and 0.17 log_10_ copies/mL lower in venous PSC vs. capillary PSC.

**Table 7:** Repeatability results.

		Standard Deviation (log_10_ titer)			Coefficient of Variation ^a^ (%)		
	Number	Mean	Median	5^th^ - 95^th^ percentile	Mean	Median	5^th^ - 95^th^ percentile
capillary PSC	64	0.09	0.08	0.02–0.25	22.9	18.1	4.0–63.4
venous PSC	65	0.08	0.07	0.02–0.18	19.9	17.1	5.7–44.5
venous DBS	43	0.11	0.09	0.03–0.30	28.5	20.7	7.1–78.3

^a^ Coefficient of Variation for log-transformed values is calculated using the following formula: $100\%\cdot \sqrt{10^{ln\;(10)\cdot \sigma^{2}}-1}$ [25]

## Discussion

The collection, storage and shipment of plasma, the preferred specimen type for HIV VL testing, requires a trained phlebotomist, centrifugation, refrigeration and/or freezing of the specimen. Under optimal conditions, HIV-1 RNA is sufficiently preserved so that the VL result accurately reflects the level of viremia in the patient. In many rural locations and especially in low- and middle-income countries, the requirements for preservation of plasma are not always available. To prevent suboptimal clinical management of people living with HIV in these areas, an alternative type of specimen is needed. While DBS are typically used in these situations, generation of a VL result that matches plasma can be compromised by the presence of viral RNA and DNA in the cellular compartment of whole blood. Furthermore, viral RNA on DBS is not indefinitely stable in the absence of refrigeration and freezing, which can partially offset the advantages over plasma.

The PSC represents an attractive alternative to DBS, since cells are filtered *in situ* and the viral RNA in the resulting dried plasma is stabilized, such that the accuracy of the VL result obtained from PSC compared to plasma is high, even under extreme conditions [13].

The results of our study confirm the high degree of correlation and agreement between VL results from PSC and plasma. We have extended previous observations by comparing PSC prepared using blood from a finger prick to EDTA-anticoagulated blood collected by phlebotomy. Our results show that VL measured in capillary and venous PSC are less than 0.15 log_10_ copies/mL (1.4-fold) different from plasma, on average; this difference is unlikely to be clinically significant. Capillary PSC VL was slightly higher than in plasma, while venous PSC VL was slightly lower; this leads to the average difference between the two types of PSC being larger (0.24 log_10_ copies/mL, or 1.7-fold). While this difference is relatively small, we speculate that the difference is related to variable amounts of fluids unique to the capillary blood collection (e.g. lymphatic fluid) or procedural issues unique to the preparation of PSC using capillary tubes. Concordance with plasma at the 1000 copies/mL threshold was over 90% for both types of PSC; misclassification was balanced in either direction for capillary PSC, while there was a tendency for venous PSC to show downward misclassification more often. Taken together, these data support the use of PSC as an alternative to plasma in situations when the preservation of plasma is not possible.

Our results using DBS were less reassuring. VL was lower in DBS compared to plasma or PSC by 0.5 to 0.6 log_10_ copies/mL or about 3- to 4-fold, on average. The concordance with plasma at the 1000 copies/mL threshold was 75%, with the majority of discordant samples having VL below 1000 log_10_ copies/mL in DBS. Our results resemble those of Hans et al. [26] in that there was excellent correlation between VL from venous PSC and plasma, but VL measurements in DBS that were above the LLOQ for DBS were lower than in venous PSC or plasma by an average of approximately 0.5 log_10_ copies/mL. The clinical implications of this finding include the possibility of mis-diagnosing (underestimating) treatment failure in patients receiving ART, which could lead to overestimation of the success of ART treatment programs, and increased risk of progression to AIDS, HIV transmission, and the development of antiretroviral drug resistance.

A repeatability study was also performed and demonstrated that HIV-1 VL measurements derived from different spots on the PSC or DBS cards prepared from the same patient are reproducible, with median %CV below around 20%. Given that spots were prepared by pipetting a defined volume of blood, and that the entire spot was used for nucleic acid extraction, this result is not surprising, and reinforces a preference for the use of microcapillary tubes for PSC preparation, as opposed to direct spotting from the finger prick.

The interpretation and generalization of our results is subject to some limitations. Comparisons between VL from plasma or PSC and DBS involved not only different specimen types, but also different VL assays (cobas HIV-1 or RealTime HIV-1). However previously published comparisons of VL quantitation in plasma by these two assays demonstrated very low average difference (cobas HIV-1 higher by average 0.1 log_10_ copies/mL) [27]. Other studies have shown good correlation between VL in plasma and DBS measured with RealTime HIV-1 [8, 19–21]. The differences in VL we observed between DBS and PSC could be related to the fact that a lower volume of blood was used in DBS (70 vs. 140 μL; both volumes chosen according to the respective instructions for use), although this would not have affected the quantitative difference we observed compared to plasma, unless there was an error in the reporting software used by the RealTime HIV assay. Another possibility could be that the longer storage time between specimen preparation and testing for DBS compared to PSC led to some degradation and lower than expected VL measurement. While some DBS were stored for more than 12 weeks, the average difference in VL compared to plasma for these specimens was not different from that of the others, although the number of specimens with VL over the LLOQ for both specimen types was low (mean DBS VL lower than plasma by 0.6 log_10_ copies/mL, n = 4). There were no cases of DBS VL < LLOQ but plasma VL >890 copies/mL. Other issues such as incomplete drying of the DBS card, insufficient desiccant use, or other errors related to sample preparation and storage could also have contributed. While exploration of these possibilities with the study site yielded no known or confirmed issues that could explain our observations, future studies could target these potential shortcomings. Finally, whether or not the cost of the PSC is offset by reduced labor or infrastructure costs at the point of collection or in the laboratory compared to DBS or plasma requires further study.

In conclusion, this field study of the practical use of PSC as a specimen type for HIV-1 VL measurement demonstrated good agreement and concordance with plasma, the standard specimen, and indicate that VL from PSC more closely matches that from plasma than VL in DBS. The use of PSC in areas where the preparation and proper storage of plasma before VL testing is challenging may be preferred over DBS, and may enable scale-up of VL testing in underserved populations.

## Supporting information

S1 Data Supplementary material including Figures A and B, and Tables A and B.
(XLSX)
